# A novel missense mutation in *ISCA2* causes aberrant splicing and leads to multiple mitochondrial dysfunctions syndrome 4

**DOI:** 10.3389/fpsyt.2024.1428175

**Published:** 2024-10-18

**Authors:** Zuhair Al-Hassnan, Mazhor AlDosary, Aljouhra AlHargan, Hanan AlQudairy, Rawan Almass, Khaled Omar Alahmadi, Saif AlShahrani, Albandary AlBakheet, Mohammad A. Almuhaizea, Robert W. Taylor, Dilek Colak, Namik Kaya

**Affiliations:** ^1^ Department of Medical Genomics, Center for Genomic Medicine, King Faisal Specialist Hospital and Research Centre, Riyadh, Saudi Arabia; ^2^ College of Medicine, Alfaisal University, Riyadh, Saudi Arabia; ^3^ Translational Genomics Department, Center for Genomic Medicine, King Faisal Specialist Hospital and Research Centre, Riyadh, Saudi Arabia; ^4^ Department of Radiology, King Faisal Specialist Hospital and Research Centre, Riyadh, Saudi Arabia; ^5^ Neuroscience Center, King Faisal Specialist Hospital and Research Center, Riyadh, Saudi Arabia; ^6^ Mitochondrial Research Group, Translational and Clinical Research Institute, Faculty of Medical Sciences, Newcastle University, Newcastle upon Tyne, United Kingdom; ^7^ National Health Service (NHS) Highly Specialised Mitochondrial Diagnostic Laboratory, Newcastle upon Tyne Hospitals National Health Service (NHS) Foundation Trust, Newcastle upon Tyne, United Kingdom; ^8^ Molecular Oncology Department, King Faisal Specialist Hospital and Research Centre, Riyadh, Saudi Arabia

**Keywords:** *ISCA2* founder variant, novel splicing variant, mtDNA, depletion, leukodystrophy, neuroregression

## Abstract

**Background:**

Iron–sulfur cluster assembly 2 (ISCA2) deficiency is linked to an autosomal recessive disorder known as multiple mitochondrial dysfunctions syndrome 4 (MMDS4). This disorder is characterized by leukodystrophy and neuroregression. Currently, most of the reported patients are from Saudi Arabia. All these patients carry a homozygous founder variant (NM_194279.2:c.229G>A:p.Gly77Ser) in *ISCA2*.

**Methods:**

We describe a patient who underwent full clinical evaluation, including metabolic, neurological, and radiological examinations. Standard genetic testing, including whole exome sequencing coupled with autozygome analysis, was undertaken, as were assessments of mitochondrial DNA (mtDNA) copy number and mtDNA sequencing on DNA extracted from blood and cultured fibroblasts. Functional workup consisted of splicing assessment of *ISCA2* using RT-PCR, biochemical assessment of complex I status using dipstick assays, and mitochondrial respiration measurements using a Seahorse XFp analyzer.

**Results:**

We present the clinical and functional characterization of a novel homozygous *ISCA2* missense variant (NM_194279.3:c.70A>G:p.Arg24Gly), leading to aberrant splicing in a patient presenting with neuroregression, generalized spasticity with exaggerated deep tendon reflexes and head lag, and progressive loss of acquired milestones. The novel variant was fully segregated in a wider family and was absent in a large control cohort, ethnically matching in-house exomes, local databases such as CGMdb and Saudi Human Genome Program, and ClinVar.

**Conclusions:**

Our analyses revealed that the variant is pathogenic, disrupting normal *ISCA2* splicing and presumably leading to a truncated protein that disturbs metabolic pathways in patient-derived cells.

## 1 Introduction

Iron–sulfur clusters (ISC or Fe–S clusters) are conserved cofactors of iron and sulfur associated with the cysteine sulfurs of proteins. These clusters are common and present in a plethora of organisms, ranging from protists to eukaryotes (1). These cofactors in eukaryotic cells are involved in diverse functions and play critical roles in many fundamental molecular processes, such as electron transfer, structural stabilization, gene regulation, and enzymatic catalysis (1). Several proteins are involved in the assembly of Fe–S clusters. Some are located in the inner mitochondrial membrane and are related to electron transport chain function, including iron–sulfur cluster assembly 1 and 2 (ISCA1 and ISCA2), which are both encoded by nuclear genes (2). Depletion of both ISCA1 and ISCA2 has been linked to incorrect assembly of ISC and biosynthesis of mitochondrial ISC (3–6). ISCA2, an essential ISC assembly member, is crucial for multiple versatile cellular mechanisms such as DNA replication, RNA transcription, oxygen sensitization, and electron transport (7, 8). The utilization of ISC occurs in complexes I–III (9, 10). Recent findings suggest that in addition to being a vital component of normal mitochondrial functionality, ISCA2 is essential for erythroid differentiation and cell proliferation (11).

Previously, we reported a homozygous pathogenic variant (a founder mutation among Saudis) of *ISCA2* in five unrelated consanguineous families comprising six infantile patients characterized by leukodystropy and neuroregression (8, 12). The syndrome is transmitted in an autosomal recessive mode of inheritance (8). Since then, the disease has been known as multiple mitochondrial dysfunctions syndrome 4 (MMDS4) (MIM Phenotype#616370; Gene/Locus MIM#615317) (13). Currently, more than 20 patients reported in the literature harbor ISCA2-related genetic defects, most of whom were from Saudi Arabia.

In this study, we report an additional case of a consanguineous Saudi family harboring the novel *ISCA2* variant. We functionally characterized this variant and compared it to previously published Saudi cases with the founder variant, detailing the clinical and genetic features of MMDS4.

## 2 Materials and methods

### 2.1 Subjects

This study was conducted at the King Faisal Specialist Hospital and Research Center (KFSHRC) using an institutional review board-approved protocol (RAC#2120022/2180004). While visiting the medical genetics clinics, the patient (whose legal guardians) and family members were asked to sign an informed written consent before donating their samples. Subsequently, they were evaluated for the project and biological specimens were collected.

### 2.2 Sample collection and nucleic acid extraction

Venous blood samples (5–10 mL) were collected in EDTA tubes from each patient and their family members. DNA was extracted from the collected blood samples using a PureGene DNA Purification Kit (Gentra Systems, Inc., Minneapolis, MN, US). DNA quality and quantity were examined on 1%–2% agarose gels and then analyzed using NanoDrop^®^ ND-1000 (NanoDrop Inc., Wilmington, DE, US).

### 2.3 Cell *c*ulture

Age-matched controls and index patient's (F1-II-2, Patient 1, Family 1) primary skin fibroblast cell lines were grown in high-glucose Dulbecco’s modified Eagle’s medium (Sigma-Aldrich, St. Louis, MO, USA) containing 10% fetal calf serum, 1× non-essential amino acids, 50-U/mL penicillin, 50-μg/mL streptomycin, and 50-μg/mL uridine at 37°C and 5% CO_2_ in a humidified incubator. For harvesting, both the medium and trypsin were prewarmed at 37°C in an incubator prior to use. The cells were then washed with 1× PBS. Depending on the flask size, sufficient trypsin was added to cover the surface of the flask, which was then incubated at 37°C for 1–2 min to dislodge the cells from the flask. Complete growth medium was added to prevent further proteolysis by quenching trypsin and maintaining the cells in suspension. To harvest the cells, they were allowed to reach 90%–100% confluency, washed with 1× PBS, incubated in a sufficient amount of trypsin, and briefly incubated at 37°C. The cells were washed again with 1× PBS and lysed with complete lysis-M (EDTA-Free) buffer (Roche, Sigma Aldrich) according to the manufacturers’ protocol. DNA and RNA extraction was performed using fibroblasts and QIAGEN kits (Venlo, Netherlands). The nucleic acid quality and quantity were determined using a Bioanalyzer 1200 (Agilent Technologies, Santa Clara, CA, US).

### 2.4 Genome-wide SNP genotyping and autozygome analysis

SNP genotyping was performed using Affymetrix Axiom Human Mapping assays on Axiom^®^ 2.0. Samples from the families were registered on Gene Titan Array plates and processed on the Titan platform (Affymetrix, Santa Clara, CA, USA). All downstream applications, such as target labeling, hybridization, array scanning, image acquisition, and quality assessment of scanned images, were performed according to Affymetrix’s protocols and guidelines. Genome-wide SNP calls were then acquired, read, and analyzed using Affymetrix Genotyping Console. Genome-wide SNPs were transferred to AutoSNPa software, and homozygous blocks, also called runs of homozygosity (ROH), were determined (14).

### 2.5 ISCA2-related Sanger *s*equencing

Quality-assessed DNA samples were used for DNA amplification. Previously tested and published *ISCA2* primers were used for the PCR (8). To assess the performance of the PCR products, amplified DNA samples were loaded and visualized on agarose gels. The amplified fragments were subjected to Sanger sequencing and analyzed using Lasergene from DNASTAR.

### 2.6 Mitochondrial DNA (mtDNA) sequencing

Genomic DNA was extracted from the fibroblasts and used as a template for mtDNA amplification. Complete genome amplification, sequencing, and analyses of sequencing results were performed as previously described (15, 16).

### 2.7 Multiple sequence alignment and protein modeling

Amino acid sequences from several species were extracted from Ensembl and aligned using the Clustal Omega software from the European Bioinformatics Institute. The sequences were aligned, and the variation site and surrounding region were targeted. Alignment and visualization were performed using the freely available Jalview and Protean tool (DNASTAR, Wisconsin, MD, USA).

### 2.8 Whole exome sequencing and variant detection

After quality assessment of the isolated DNA, sequence capture and library preparation were performed using SureSelect assays. The libraries were then sequenced on HiSeq 2000 (Illumina, San Diego, CA, USA) to obtain a minimum of 30-fold coverage. Sequences were aligned, and the resulting variant call format (VCF) file, which contains 55,091 variants, was filtered and analyzed using publicly and commercially available software and tools.

### 2.9 Assessment of mtDNA copy number using quantitative PCR (qPCR)

Depletion assays were carried out using *NALCN* (nuclear marker) and *ND1* (mitochondrial gene) on DNA extracted from the index patient’s cultured fibroblasts, as previously described (8). The delta delta C_T_ method was used for the copy number calculation.

### 2.10 Dipstick assay for complex I

Dipstick assay kit designed to measure complex I activity (MitoSciences/Abcam, Cambridge, UK) was utilized as done previously (8). Fibroblasts from index patients and controls were used in the experiments. The manufacturer’s protocols were strictly followed throughout the experiments.

### 2.11 RT-PCR analysis of the novel variant

High-quality and undegraded RNA was used for cDNA synthesis using a High-Capacity cDNA Reverse Transcription Kit (Applied Biosystem Corp., Waltham, MA, USA). For the primers used in RT-PCR, the Primer3 web tool was utilized for exon spanning primers to ensure successful and exclusive amplification of exonic regions without intrusion of genomic sequences. The expression of *ISCA2* was compared to that of *GAPDH* (F-primer 5′-3′ CTGCCAACGTGTCAGTGGTG and R-primer 5′-3′ TCAGTGTAGCCCAGGATGCC) in the same cells.

### 2.12 Mitochondrial respiration measurements

We used Seahorse XFp analyzer (Agilent) to measure oxygen consumption rate (OCR) to determine key parameters of cellular metabolism using control and patients’ cells as described (17–19). One day prior to the assay, fibroblasts (20,000 cells/well) were seeded in a Seahorse 8-well mini-culture plate with growth medium (MEM supplemented with 10% FBS, 1% penicillin–streptomycin, and 1% L-glutamine), placed in a 37°C incubator with 5% CO_2_ and allowed to adhere overnight. The sensor cartridge was hydrated overnight in an XF calibrant at 37°C in a non-CO_2_ incubator. The next day, the cells were washed twice with warm assay medium, and 180 µL of assay medium was added per well. The cell culture mini-plate was then placed in a 37°C non-CO_2_ incubator for 45–60 min prior to the assay. Each sample was measured in duplicates (technical replicates). The results were calculated and produced using the Seahorse Wave software version 2.6.

### 2.13 Statistical analysis

All statistical analyses were performed using GraphPad Prism v6.0 (GraphPad Software, San Diego, CA, USA). Data are expressed as mean ± standard error of the mean (SEM). To compare the means between two groups, an unpaired Student’s t-test was performed, with Welch’s correction, if applicable. All statistical tests were two-sided, and a p-value <0.05 was considered statistically significant.

## 3 Results

### 3.1 Clinical findings

The details of the clinical examination results are presented in **Table 1**.

**Table 1:** Clinical details of the singleton carrying the novel *ISCA2* variant.

Sex		Male
Age at presentation (months)		8
Current status (alive or dead)		unknown
Consanguinity		Yes
Family history of similar illness		No
Ethnicity		Arab
Mutation	cDNA change	ISCA2: NM_194279.4: c.70A>G
	Amino acid change	p.R24G
	Mutation type	Missense and aberrant splicing
	Zygosity	Homozygous
Neuromuscular findings	Developmental milestones loss	+
	Motor regression	+
	Hypertonia	+
	Seizure	–
	Hyperreflexia	–
	Ataxia	–
	Cognitive disability	+
	Spasticity	+
	Speech impairment	+
	Hearing impairment	+
	Loss of sight	+
	Optic neuropathy	+
	Visual fixation and following loss	+
	Nystagmus	–
Neuro-imaging, MRI	White matter abnormality	+
	Gray matter abnormality	+
General features	Non-bilious vomiting	–
	Irritability	+
	Sleeplessness	+
	Startle reflex	+
	Head lag	+
Metabolics	Lactic acidemia	–
	Hyperammonemia	–

+ = Yes, − = No.

The patient was referred to our center at the age of 24 months for evaluation of neuroregression. He was a product of a full-term pregnancy with normal spontaneous vaginal delivery and an uneventful perinatal history. He was well until the age of 8 months, when he started to have irritability and poor eye contact. He progressively lost his acquired milestones and became spastic, with an inability to sit, hold objects, or babble. There was no history of seizures or abnormal movement. The parents were consanguineous. There were five other children who were alive and well. Physical examination revealed that both head circumference and height were at the 5th percentile, while weight was just below the 5th percentile. He was irritable and unable to fixate or follow. No dysmorphic features or skin stigmata were observed. He had generalized spasticity with exaggerated deep tendon reflexes and head lag. Organomegaly was not observed. Eye examination revealed a diffuse pallor of the optic disc.

#### 3.1.1 Neuroradiological findings

**Figure 1:**
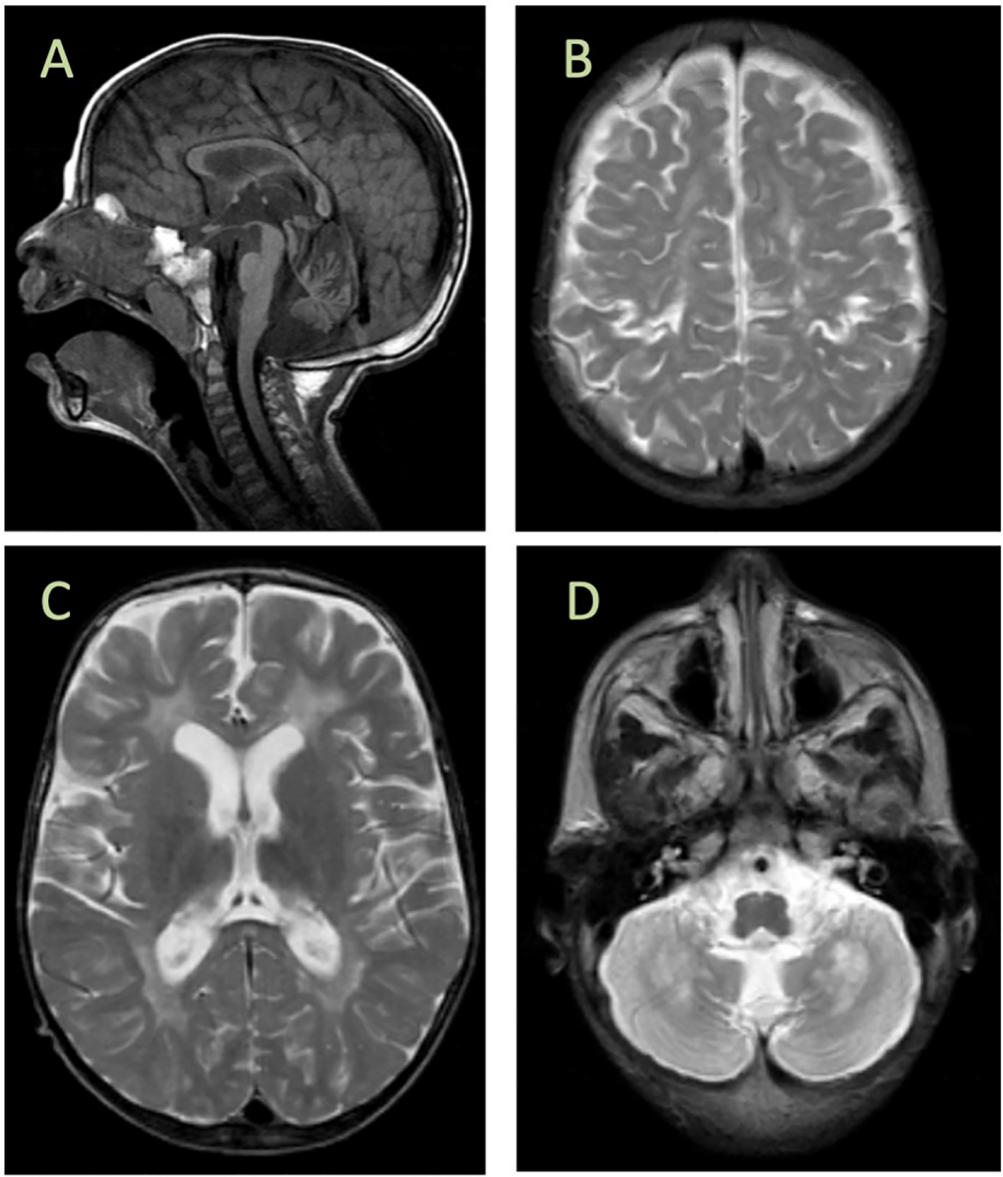
Brain MRI findings. **(A)** Cerebellar atrophy and decreased signal in the corpus callosum, diffuse increased signal, and cystic encephalomalacia in the white matter **(B, C)** with bilateral involvement of the gray matter around the central sulcus **(D)**. MRI was performed at age of 2 years.

Brain MRI showed confluent involvement of the white matter in the supratentorial brain parenchyma spanning from the periventricular to the subcortical area. It involves the corticospinal tracts in the posterior limbs of the internal capsules, tegmentum of the middle cerebral peduncles, and dorsal aspect of the brain stem. The white matter changes extended to the posterior structures, specifically the peridentate white matter of the cerebellar hemispheres. Diffuse atrophy of the corpus callosum is also observed. The brain MRI findings are shown in **Figures 1A–D**.

#### 3.1.2 Molecular genetic findings

**Figure 2:**
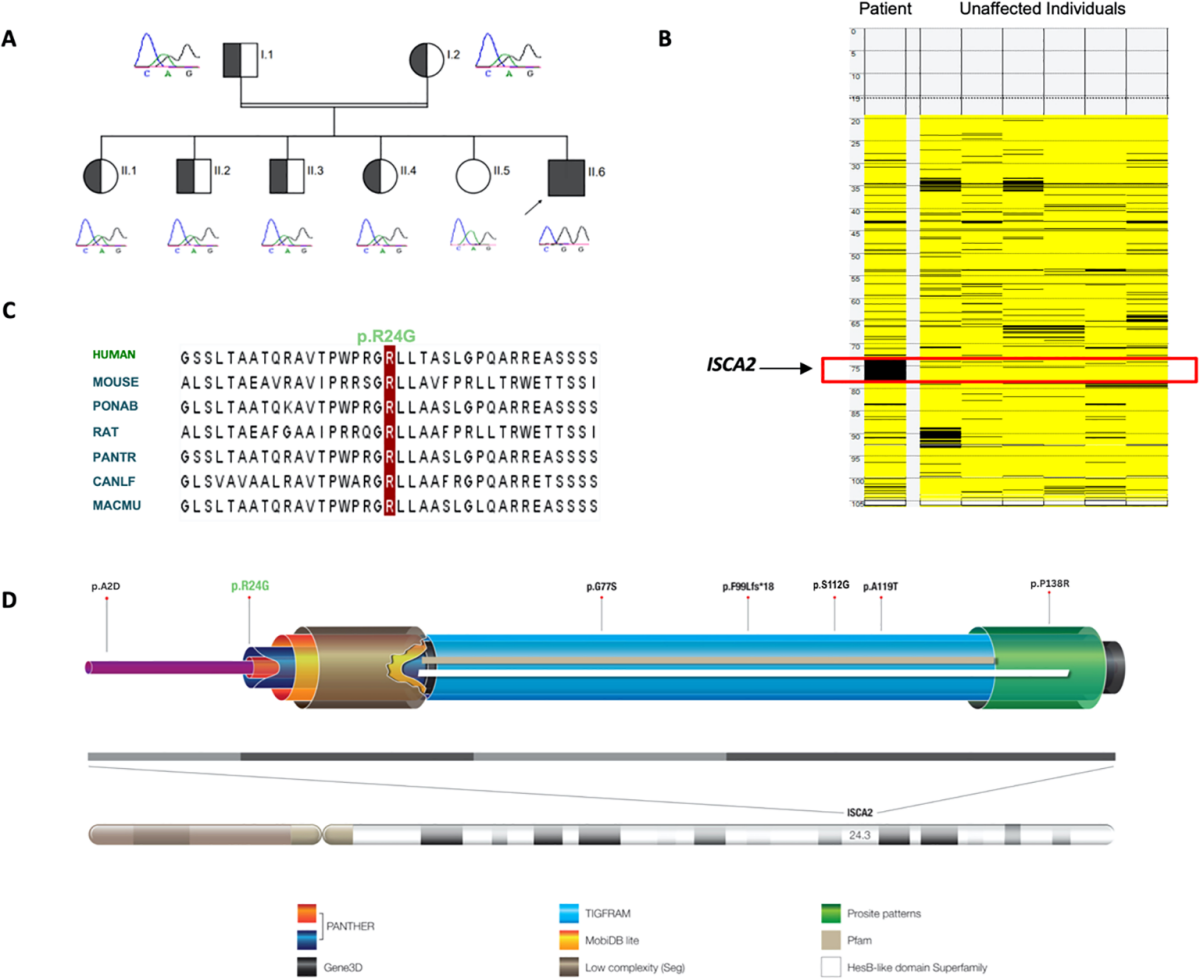
**(A)** Pedigree showing the affected individual and carriers of the novel and pathogenic *ISCA2* variant c.70A>G; p.Arg24Gly and full segregation of the phenotype in the family, indicating a recessive inheritance pattern. Short Sanger sequencing images are presented below. **(B)** Autozygome analysis of the patient and unaffected individuals revealed a unique homozygosity block on the long arm of chromosome 14 in the affected individual. **(C)** Alignment of amino acid sequences at position 24 (as indicated by the red column) within ISCA2, showing high conservation in different species. **(D)** Structure and localization of the human ISCA2: The *ISCA2* gene is located on chromosome 14q24.3 and consists of four exons shown as light and dark gray boxes (bottom). The ISCA2 protein has one essential functional domain for iron–sulfur (Fe–S) biogenesis, which overlaps with the predictions of PANTHER, Gene3D, TIGRFAM, MobiDB lite, Prosite pattern, Superfamily, and Pfam protein databases. Local complexity in amino acid sequences is also represented as ‘low complexity segments domain. The novel *ISCA2* mutation site and previously reported mutations in *ISCA2* are indicated (top panel).

Previously, we reported six patients with MMDS4 and later expanded the phenotypic spectrum of the disease in our country (8, 20). Most reported cases were from Saudi Arabia, and the first reported mutation (NM_194279.2:c.229G>A:p.Gly77Ser) is an ancient founder that appears in the central and western regions of the country, and we expected to encounter more cases in this region. Hence, we continuously searched for additional patients with this disease. Interestingly, we encountered a singleton from a consanguineous Saudi family whose genomic DNA was comprehensively subjected to diagnostic and research-based genetic testing, including whole mitochondrial DNA (mtDNA) sequencing. After exhaustive filtering of whole-exome sequencing data, the analysis revealed a missense and homozygous variant (NM_194279.3:c.70A>G:p.Arg24Gly) in *ISCA2*. The filtering approach and criteria were performed using previously reported protocols (21–27). The variant was fully segregated in the family members (**Figure 2A**; **Supplementary Figure 1**) using Sanger sequencing. Moreover, our autozygome analysis revealed that *ISCA2* sits in a long stretch of a homozygous run of homozygosity (ROH) block on chromosome14q24.3 specific to the affected individual (**Figure 2B**). We then searched our in-house exomes and local databases such as the Saudi Human Genome Database (SHGP) and King Abdullah International Medical Research Center (KAIMRC) Genomic Database (KGD), as well as ethnically matched control data sets (totals to more than 2,703) that did not have any positive match in the homoallelic state. This variant was not encountered in ClinVar and was classified as PP3 (Strong), PM2 (Supporting), or PB1 (Supporting) based on the American College of Medical Genetics and Genomics (ACMG) criteria. The variant was also not reported in GnomAD in the homozygous state (allele frequency: 0.000003554; allele count: 5; number of homozygotes: 0).

**Figure 3:**
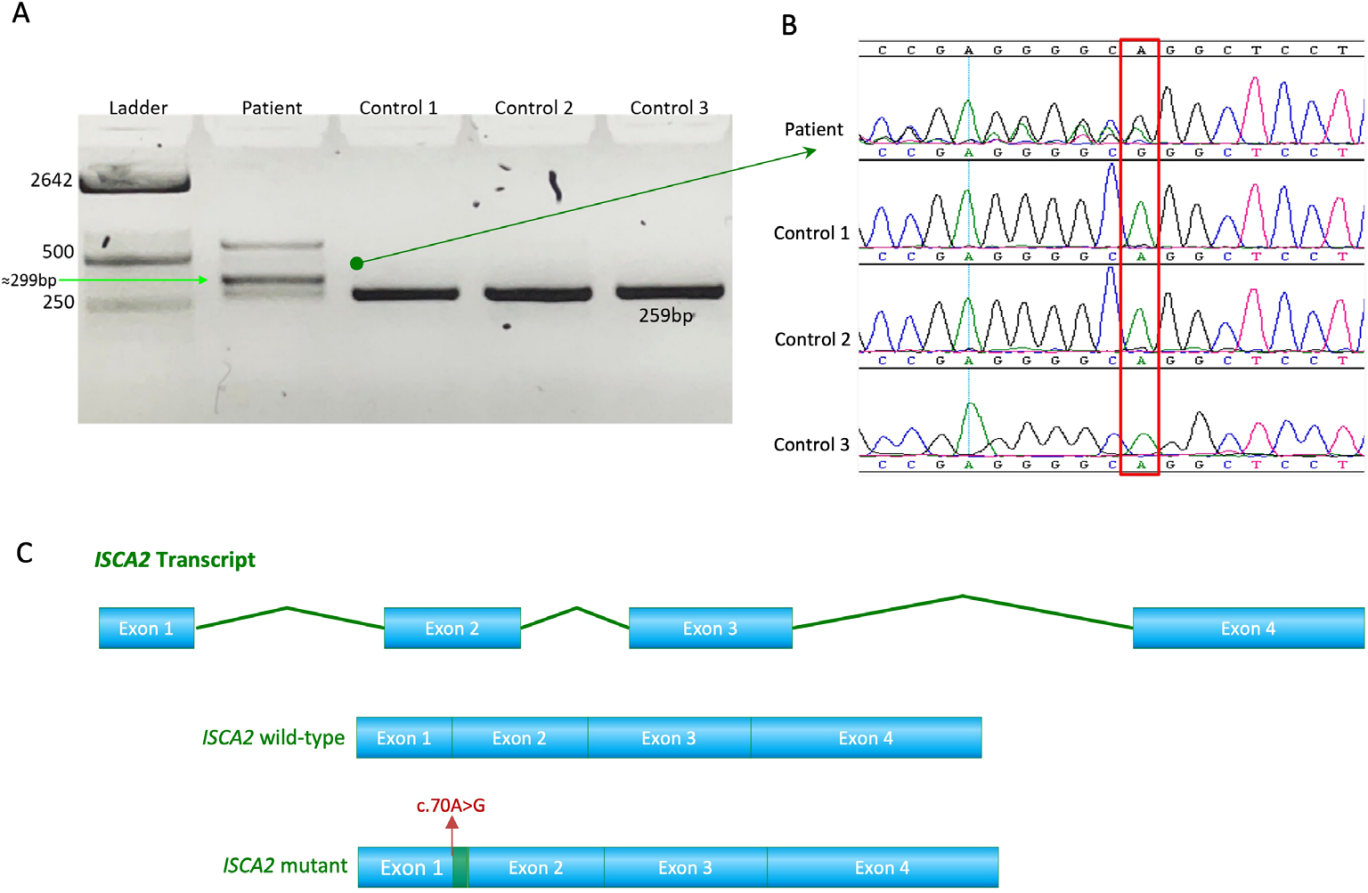
**(A)** RT-PCR analysis of RNA extracted from the patient’s fibroblasts showed faulty splicing due to the transition compared to the controls. **(B)** Sequence chromatogram showing the novel and pathogenic *ISCA2* variant c.70A>G; p.Arg24Gly in the RNA extracted from the patient's fibroblasts. **(C)** Transcript maps of *ISCA2* showing the normal transcript vs aberrant transcript. The novel variant, the aberrant transcript, causes faulty splicing, resulting in a retention of RNA transcript.

The novel variant (c.70A>G) in *ISCA2* replaces an A to G, leading to a change from arginine to glycine in the 24th amino acid position that sits in proximity to the splicing site on a highly conserved region and important domain (**Figures 2C, D**). After searching the frequency of the variant in a wide range of databases to ensure its novelty, the variant’s functionally deleterious potential was investigated using various experimental approaches. Initially, because the variant is close to the exon/intron junction, we suspected that it may cause a splicing error. To test this hypothesis, we used total RNA samples extracted from the index patient fibroblasts as well as those of the control samples (n = 3) and performed RT-PCR analysis. This experiment revealed multiple bands (n = 3) that were displayed on an agarose gel (2%) in comparison to those of the controls (**Figure 3A**). Sequencing of the bands revealed a normal product (~250 bp), an alternative splicing product (~300 bp), and a non-specific PCR band (~560 bp) (**Figures 3A, B**). Normal and aberrant transcript maps are shown in **Figure 3C**. All three bands consistently appeared in the patient’s sample, although several optimization attempts were made using different primer sets to remove the upper band. Sequencing of the RT-PCR product showed retention of the intronic sequence presumably leading to protein truncation (**Supplementary Figure 2**).

**Figure 4:**
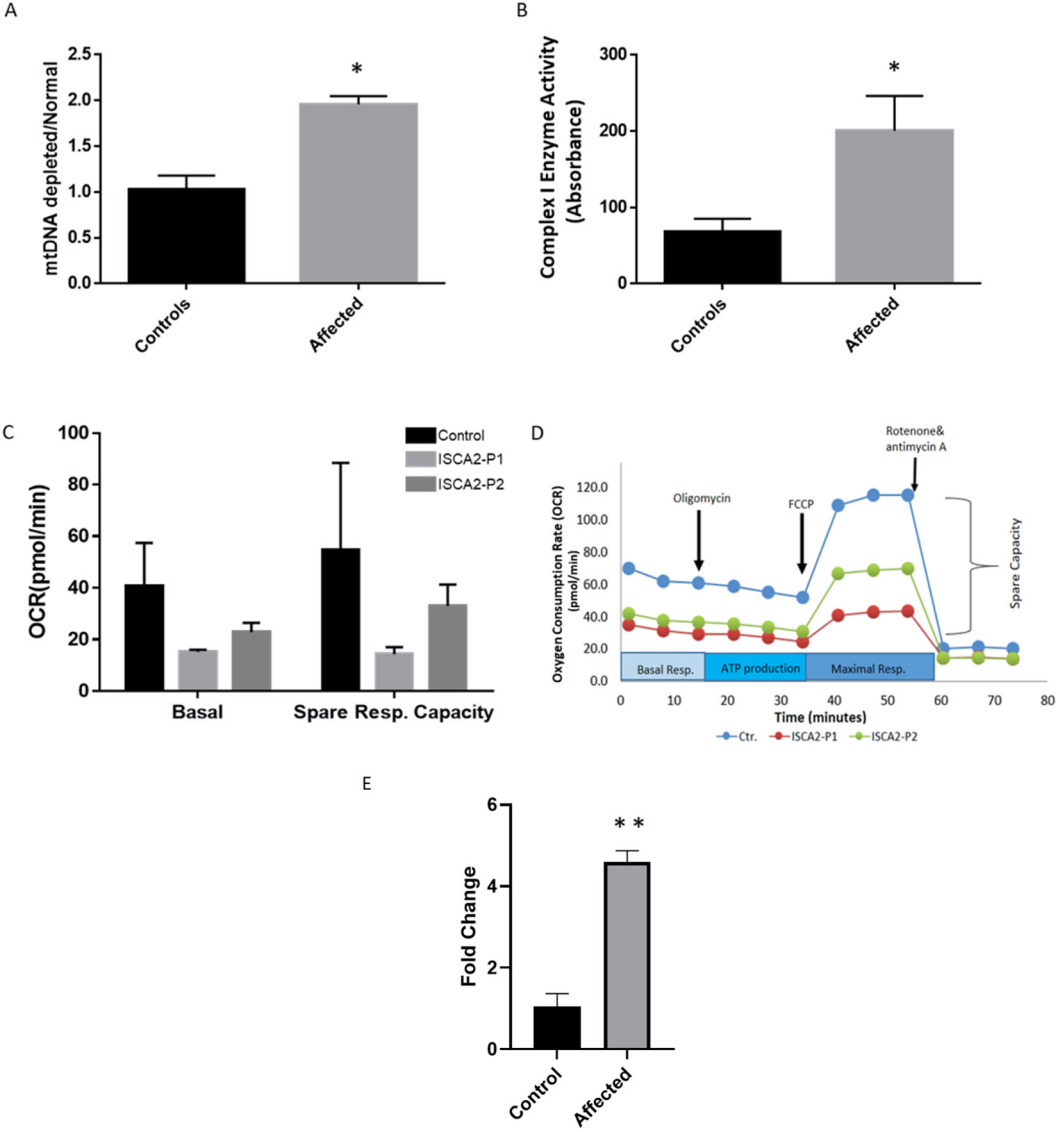
**(A)** Real-time PCR analysis of individual and control fibroblasts to assess mtDNA depletion. The assay indicated increased levels of mitochondria in the patient’s fibroblasts compared with the controls. The data represent the mean ± standard error (SE) of three controls and one patient (each sample was run in triplicate). The patient showed a significant increase compared to the controls. **(B)** Complex I enzyme activity dipstick assay. The results showed considerable evidence of mitochondrial respiratory chain abnormalities in fibroblasts from the index patient compared with controls. The data represent the mean ± standard error (SE) of four controls and one patient (each sample was run in triplicate). The patient showed a significant increase compared to the controls (p <0.05). **(C, D)** Seahorse XF cell mitochondrial stress test profile showing mitochondrial respiration parameters measured in fibroblasts from control subjects and two patients. Oxygen consumption rates measured in healthy subjects and individuals with ISCA2 deficiency. Spare respiratory capacity is the difference between basal and maximal respiration and measures the ability of cells to respond to increased energy demand. ISCA2-P1 was the index patient carrying the novel variant reported in this study, whereas ISCA2-P2 was another patient with a homoallelic missense founder mutation (NM_194279.2;c.229G>A;p.Gly77Ser). Two replicates were used for each experiment. Data are presented as the mean ± SEM. Both mitochondrial basal respiration and spare respiratory capacity were reduced in both patients compared to the control; however, the difference was not statistically significant. **(E)** Quantitative RT-PCR was performed on RNA extracted from the index patient fibroblasts and compared with that of the control fibroblasts. The results showed significantly increased expression levels of ISCA2 in cultured fibroblasts of the index case. The data represent the mean ± standard error (SE) of four controls and one patient (each sample was run in triplicate). The patient showed a significant increase compared with the control group (p <0.0001). In the figures, black indicates the control(s) and light gray indicates the affected individual(s). Two-tailed Student’s t-tests with Welch’s corrections were performed; *p <0.05 and **p <0.0001 indicate statistical significance.

Next, we sought to determine whether the variant led to any depletion of the cultured fibroblasts in the index case. As previously described (8), we utilized a qPCR approach to investigate mitochondrial copy number changes. The results revealed increased mtDNA copy number in the patient’s fibroblasts (**Figure 4A**).

#### 3.1.3 Biochemical findings

Next, we used a complex I enzyme activity dipstick assay to investigate the functional consequences of this variant and the likely deficiency in fibroblasts from the patient. Dipstick tests revealed increased levels of complex I activity in fibroblasts from patients compared to those from the controls (**Figure 4B**).

To gain further functional insight into the likely consequences of the c.70A>G novel mutation, we performed a mitochondrial respiration test on fibroblasts from the control and two patients, our index patient and another patient who carried the homozygous missense founder mutation (NM_194279.2:c.229G>A:p.Gly77Ser). Both patients’ cells showed a decrease in mitochondrial basal respiration and spare-respiratory capacity as compared to those of the control (**Figures 4C, D**). Moreover, quantitative RT-PCR results showed a significant increase in the expression levels of ISCA2 in the cultured fibroblasts of the index case compared to those of the controls (p <0.0001) (**Figure 4E**).

## 4 Discussion

The first *ISCA2* human mutation (NM_194279.2 c.229G>A; p.Gly77Ser), a homozygous missense founder, was identified in Saudi patients belonging to unrelated consanguineous families. Additional disease-causing variants have been discovered over the past few years. Among these, the most commonly encountered mutation is the Saudi founder variant. The total number of MMDS4 patients reached 44, mostly with the Saudi founder mutation (n = 37) except for six non-Saudis. Subsequent cases have been reported in non-Saudi Arab patients with different types of mutations. For example, an Italian patient was identified to carry a different mutation: a paternal frameshift (c.295delT:p.Phe99Leufs*18), resulting in a premature stop codon and a maternal missense variant (c.334A>G:p.Ser112Gly) with compound heterozygosity (30). Moreover, two unrelated patients of French origin were identified as carrying homozygous missense variants in *ISCA2* (c.154C>T, p.Leu52Phe and c.313A>G, p.Arg105Gly) that resulted in reduced activity of complex I (28). More recently, an Iranian patient was found to have an *ISCA2* variant (c.355G>A, p.Ala119Thr) (29).

Here, we present the clinical and functional characterization of a novel homozygous *ISCA2* missense variant (NM_194279.3:c.70A>G; p.Arg24Gly) leading to aberrant splicing. This patient presented with neuroregression, generalized spasticity with exaggerated deep tendon reflexes, and head lag with progressive loss of acquired milestones that started with irritability and poor eye contact. The clinical presentation of the patient was consistent with the ISCA2-related mitochondrial disease phenotype (8, 13, 21). This study identified the second Saudi ISCA2 variant among Saudi Arabs, expanding the genotypic spectrum of MMDS4 even in a highly consanguineous population, indicating that more cases remain to be identified.

The novel transition, as predicted by SpliceAI, causes a splicing error due to its location. We investigated this using RT-PCR on the total RNA extracted from patient fibroblasts. RT-PCR revealed faulty splicing due to this transition. The splicing error caused retention of the part of the intronic sequence, resulting in a larger transcript compared to control transcripts (**Figures 3A–C**).

In addition, this novel mutation caused increased mtDNA copy number and may also lead to increased complex I activity in the patient’s fibroblasts. However, we were unable to confirm this increase in activity using another method. Moreover, we could not compare citrate synthase activity to complex I activity; therefore, our results in this regard are not conclusive. Since ISCs are known to play a major role in oxidation–reduction reactions for mitochondrial electron transport, mainly complex I and complex II of oxidative phosphorylation (1, 30), this novel mutation is expected to cause such aberrant activity, as other studies have shown (6, 28). Using the Seahorse approach, our assessment of mitochondrial respiration revealed a decrease in the mitochondrial respiration parameters in the patients’ cells compared to those of the control. Similar findings were reported in a previous study of two patients with a founder homozygous missense mutation (NM_194279.2 c.229G>A; p.Gly77Ser) in *ISCA2*, which showed an increase in the activity of complex I and decreased mitochondrial respiration (31). The likely mitochondrial respiration deficiency observed in our patient may indicate the involvement of this novel mutation in pathogenicity by affecting electron transport through the mitochondrial respiratory chain complexes.

Notably, this novel variant increased both the mtDNA copy number and mRNA expression in our index patient, as opposed to the founder homozygous missense mutation (NM_194279.2 c.229G>A:p.Gly77Ser), which showed decreased levels (8, 31). This increase as well as the possible increase in complex I activity in our patient, may be attributed to a compensatory response triggered by a defect in ISCA2 function because of this variant. Moreover, mitochondrial mass was found to be increased in fibroblasts due to a defect in the respiratory chain (32), which could also explain the increased mtDNA copy number in patient fibroblasts.

Generally, the affected individuals with MMDS4 are characterized by neurodegeneration, developmental regression, failure to thrive, quadriplegia, truncal hypotonia, optic atrophy, and leukoencephalopathy (8, 13). The c.229G>A (p.Gly77Ser) variant caused decreased oxygen consumption and overall respiration, ATP production, and mitochondrial membrane potential, and significantly decreased complex II and minimal reduction in complex IV (31). Interestingly, an increase was also observed for complex I subunit NDUFB8. Further investigations also pointed out the negative impact of the variant on the functioning of proteins, specifically for 4Fe–4S clusters, but not 2Fe–2S proteins (29). However, a patient with bi-allelic variants p.Ala2Asp and p.Pro138Arg was found to have a milder phenotype with a longer life span, better psychomotor function, and cavitating leukodystrophy (30).

Taken together, these data indicate that this novel variant causes pathogenicity in our patient; however, it is important to note that technical issues related to the maintenance of patient-derived cells prevented us from performing confirmatory functional studies.

## 5 Conclusion

Here, we report the first case of a variant other than the founder mutation in Saudi Arabia. Our study reports the second Saudi *ISCA2* variant among Saudi Arabs. This study expands the genotypic spectrum of MMDS4, even in a highly consanguineous population in which a founder variant is frequently encountered in medical genetics clinics, considering that the disease is a rare disorder. MMDS type 4 is still more common in Saudi Arabia than in any other population studied to date.

## Data Availability

The datasets for this article are not publicly available due to concerns regarding participant/patient anonymity. Requests to access the datasets should be directed to the corresponding author.

## Supplementary material

Supplementary Figure 1 Sanger sequencing results of the PCR with novel *ISCA2* variant.

Supplementary Figure 2 Aberrant transcript presumably leads to a protein truncation.
