# Supplementary figures and images for: A novel missense mutation in ISCA2 causes aberrant splicing and leads to multiple mitochondrial dysfunctions syndrome 4
Source: Front Psychiatry. 2024 Oct 18;15:1428175. doi: 10.3389/fpsyt.2024.1428175 (PMC11561297; doi:10.3389/fpsyt.2024.1428175)

SUPPLEMENTARY FILE 1:

SANGER SEQUENCING RESULTS/PCR

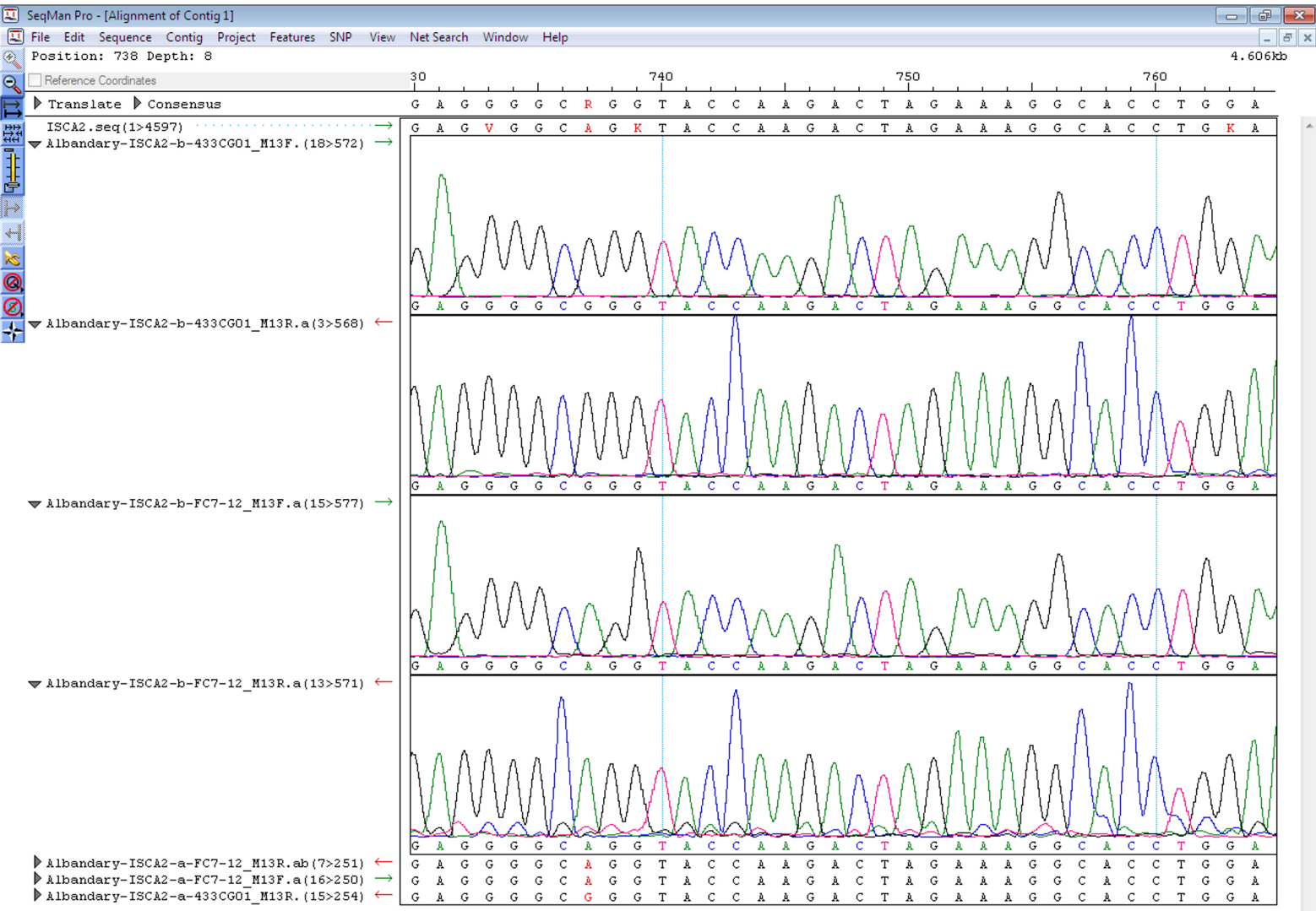

Supplement: Supplementary Figure 1 — Sanger sequencing results of the PCR with novel ISCA2 variant. [file Image1.pdf]

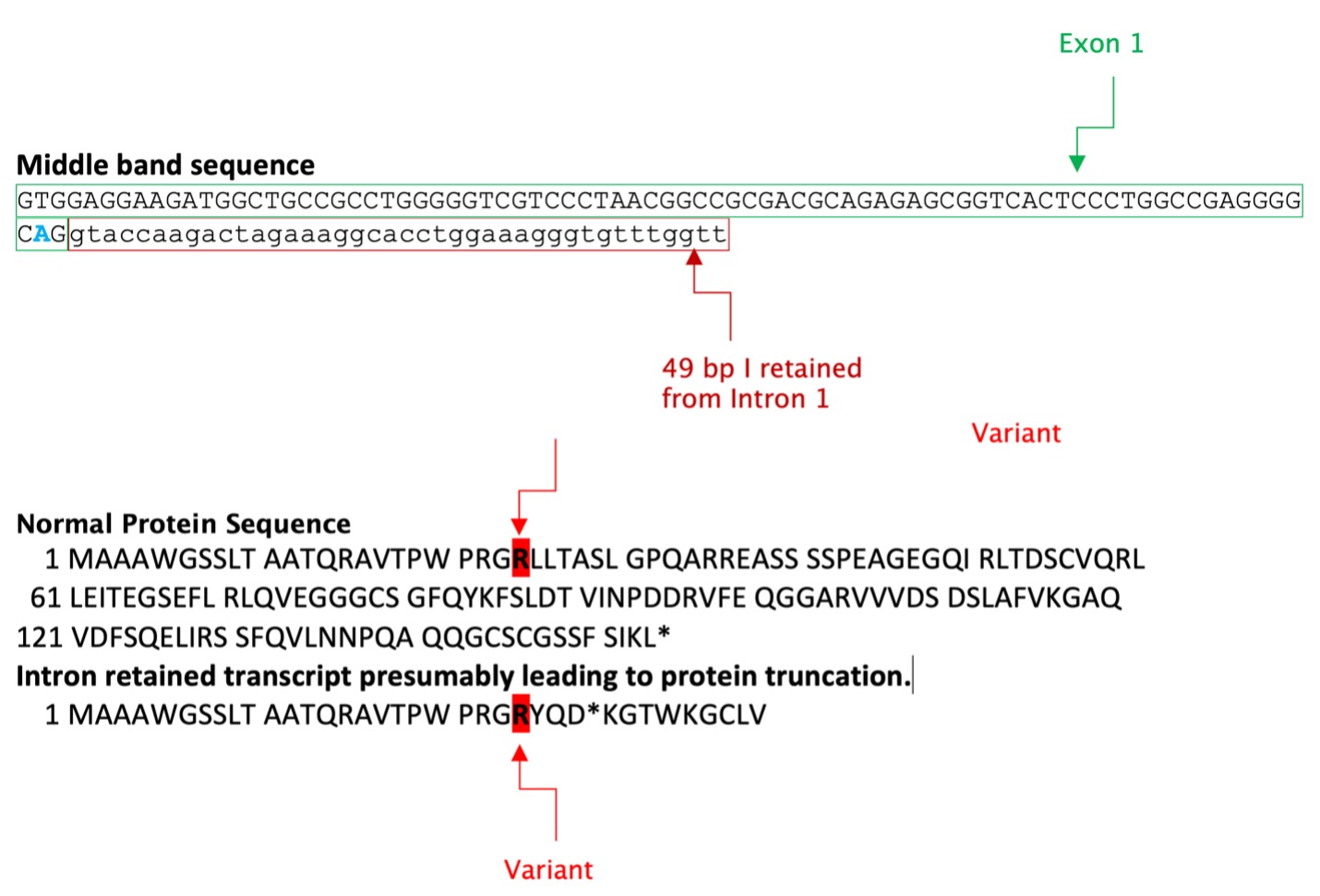

Supplement: Supplementary Figure 2 — Aberrant transcript presumably leads to a protein truncation. [file Image2.jpeg]
